# Hospitalization costs and length of stay of Japanese children with respiratory syncytial virus: A structural equation modeling approach

**DOI:** 10.1097/MD.0000000000011491

**Published:** 2018-07-20

**Authors:** Rosarin Sruamsiri, Hiroshi Kubo, Jörg Mahlich

**Affiliations:** a Health Economics, Janssen Pharmaceutical KK, Tokyo, Japan; b Center of Pharmaceutical Outcomes Research, Naresuan University, Phitsanulok, Thailand; c Research and Development Department, Janssen Pharmaceutical KK, Tokyo, Japan; d Düsseldorf Institute for Competition Economics (DICE), University of Düsseldorf, Düsseldorf, Germany.

**Keywords:** economic burden, health care costs, hospitalization costs, Japan, length of stay of Japanese children, prevention, respiratory syncytial virus, respiratory syncytial virus treatment, structural equation modeling, vaccine

## Abstract

**Background:**

This study sought to identify factors that impact the total health care costs associated with hospitalization of young Japanese children with respiratory syncytial virus (RSV).

**Methods:**

Children admitted between April 2014 and March 2015 with at least a confirmed diagnosis of RSV and 2 days of hospital stay were considered for inclusion. Data analyses of hospital claims were performed using a structural equation modeling approach.

**Results:**

A total of 6811 Japanese inpatients (<5 years old) diagnosed with RSV were included. The average length of stay was 7.5 days with a mean total health care cost of US Dollars (USD) $3344 per hospitalization. Intensive care unit hospitalizations were associated with greater costs (USD +$4951) compared to routine hospitalizations. The highest procedure-related cost drivers were blood transfusions (USD +$6402) and tube feedings (USD +$3512).

**Conclusion:**

The economic burden of RSV-related infection hospitalizations in Japan is considerable. Efforts should be toward immunization and therapeutic treatment strategies that reduce severity, prevent, or reduce the duration of hospitalization.

## 1 Introduction

Respiratory syncytial virus (RSV) is a highly prevalent and contagious virus that often results in lower respiratory tract infections which may cause other serious complications. RSV is the primary cause of hospitalization among infants with almost all experiencing their first RSV infection by the age of 2.^[1–3]^ In developed countries 1% to 3% of all children with RSV infection are hospitalized.^[4]^ According to the latest epidemiologic study, the mortality from complications due to this disease stands at close to 118,200 cases annually (uncertainty range 94,600–149,400).^[5]^ Moreover, the study reported a significant burden of RSV infection in neonates with an annual occurrence of nearly 40 episodes per 1000 neonates. Collectively, these data together with the significance of the disease in the <6 months age group indicate development of protective measures such as immunization or therapeutics against the RSV infection at birth or treatments to reduce severity of RSV infection would be highly desirable to control the disease.^[5]^

There are 2 subtypes of RSV namely type A and type B, with differences in the envelope proteins on the viral shell. While both subtypes of the virus are equally infectious, emerging evidence indicates that type A may result in more severe disease.^[6]^ Other associated risk factors include young age, premature birth, passive smoke exposure, lack of breastfeeding, chronic lung disease, and congenital heart disease.^[7–10]^ Children <2 years of age often present with initial clinical symptoms of cough, mild fever, and rhinorrhoea which develop into wheezing, tachypnea, crackles, as well as increased work of breathing. Also, the infant may exhibit characteristics of poor feeding, vomiting, or irritability. Despite significant improvement in most of the affected children within 3 to 5 days, those with underlying comorbidities such as immune or chronic lung disease may progressively become worse.^[11]^ The infection caused by RSV is a seasonal disease; in temperate climates, the RSV activity typically peaks during the winter months,^[12]^ while in tropical climates, RSV infection tends to peak during the rainy season and the hottest months.^[13]^

There are considerable costs associated with RSV-related infections. In 2000, close to 98% of all hospitalizations related to RSV infections in the United States were reported in children <5 years of age. In addition, hospitalizations due to RSV-related infections (US Dollars [USD] $394 million) together with all other medical encounters (USD $258 million) for these children were an estimated USD $652 million of the total annual medical costs.^[14]^ The average cost of RSV-related hospitalization was an estimated USD $14,832 annually for all infants. Furthermore, the health care costs of RSV-related hospitalization in the United States for high-risk infants up to 1 year of age are reportedly from USD $20,160 to $39,399 annually.^[15]^ At the same time, the average costs of intensive care unit (ICU) admissions were an estimated USD $35,000 to $89,000 for RSV-related hospitalizations with higher costs for younger infants (<90 days old) compared to the older infants.^[16]^ While the cost of RSV-related hospitalization for infants <6 months in Canada was CAN $23,030,^[17]^ in China this health care cost was USD $571.8 (US$ 909.6 for ICU admission).^[18]^ In addition, results of multivariable logistic regression demonstrated that children > 6 months of age and with comorbidities such as chronic lung disease had higher hospitalization costs when compared to those ≤6 months of age.^[18]^

While a previous study in Japan reported hospitalization of a significant number of children <3 years of age (31.4%) with RSV-related infections, to date there are no published studies on the economic burden of RSV-related health care utilization in Japan. Therefore, given that in recent years the number of new RSV cases reported is at all-time high,^[19]^ the purpose of this study is to determine the cost of health care associated with RSV-related hospitalization using structural equation modeling (SEM) approach.

## 2 Methods

### 2.1 Patient selection

We utilized a commercially available hospital claims data bank from Medical Data Vision Co Ltd (MDV). This is a national administrative database of approximately 4,400,000 patients that represent approximately 3% of the total Japanese population.^[20]^ Previously, the MDV database has been used to examine a wide range of medical conditions in Japan such as rheumatoid arthritis,^[21–23]^ schizophrenia,^[24]^ infectious diseases,^[25]^ multiple sclerosis,^[26]^ hypertension,^[27]^ or prostate cancer.^[28,29]^ We considered the inpatient claims from patients with hospital admission between April 1, 2014 and March 31, 2015 and at least 1 confirmed RSV-related diagnosis (International Classification of Diseases 10th Revision codes: J12.1, J20.5, J21.0, and B34.8) as well as a minimum hospital stay of 2 days (confirmed by at least 1-night stay in the hospital). We limited our analysis to patients of up to 5 years of age. We performed subgroup analysis for the following age groups: <1, 1, 2, and 3 to 5 years.

### 2.2 Calculations of hospitalization associated costs

Total health care cost (total hospitalization cost) comprised all costs of health care services incurred during each hospitalization period. These included basic management fees, examination, diagnostic and medical procedures, and medications. In addition, Diagnosis Procedure Combination (DPC) cost (which is a case-mix reimbursement cost) was defined as the insurance reimbursed costs. All costs were converted from Japanese yen (JPY) to USD according to the average exchange rate during the April 2014 to March 2015 period (Financial Market Department, Bank of Japan; 1 USD = 109.33 JPY).^[30]^

### 2.3 Statistical analysis

**Figure 1:**
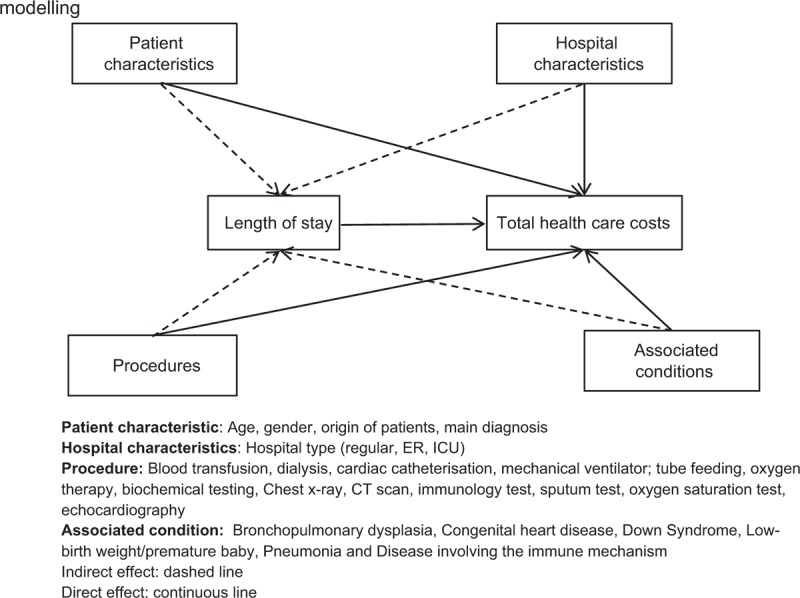
Path showing the relationship between each variable using structural equation modeling.

Descriptive analyses were performed on baseline characteristics as well as resource use, length of stay (LOS) and total health care cost. Since LOS is usually an important driver of the total hospitalization costs,^[31,32]^ we considered a SEM approach to assess the relationship between the characteristics of patients, hospital procedures as well as LOS, and hospitalization costs by considering LOS as an intermediate effect. SEM is a flexible multivariate statistical framework with a wide range of applications that can be used to model complex relationships between variables.^[33]^ The SEM framework allows assessment of relationships among variables by integrating the strengths of factor analysis and multiple regression in a single model that can be tested statistically.^[34]^ More specifically, in this study, we performed a path analysis which is a special case of the SEM framework that allows an exploration of the causal links (direct and indirect effects) between exogenous variables and one or more endogenous variables. In this framework, the total effects of a covariate on the main dependent variable can be divided into 2 categories of effects: the indirect effects, consisting of the effect of the covariate on one or more intermediary endogenous variables, which in turn translates into an effect on the main variable; and the direct effect, which is the remaining effect of the covariate on the main variable while controlling for their indirect effects.^[35]^ In our analysis, the main endogenous variable of interest was the total hospitalization cost expressed in Japanese yen. The independent variables were assumed to have both a direct effect on total hospitalization costs and indirect effects through the LOS. The relationship between each variable is depicted as a flow diagram in Figure 1. Statistical analyses were performed using Stata 15.0 (StataCorp LLC, College Park, Taxas).^[36]^

## 3 Results

**Figure 2:**
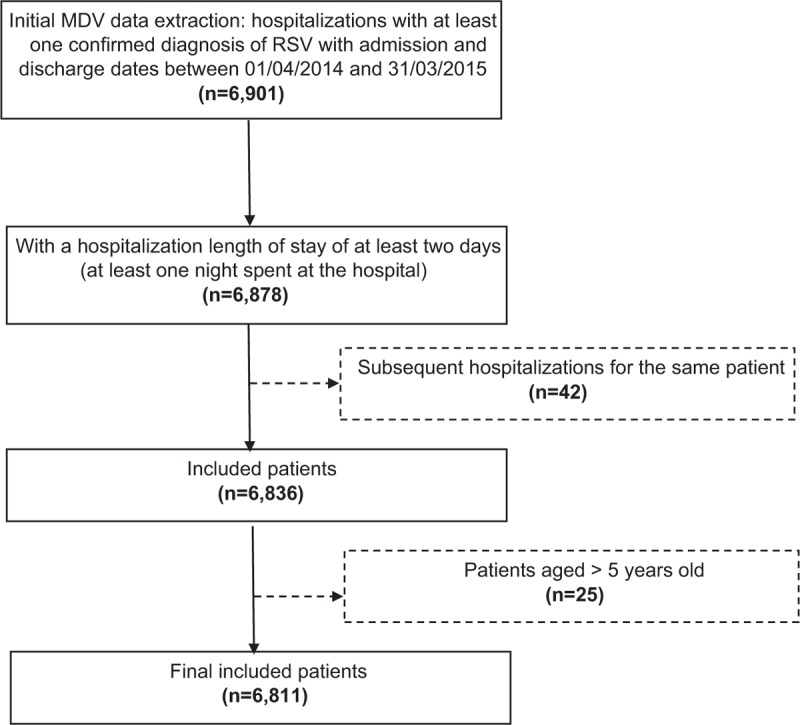
Patient flowchart.

The final analysis included a total of 6811 Japanese children <5 years old hospitalized with RSV-related infections. Patients >5 years (n = 25) were excluded from the analysis. We also excluded 42 rehospitalized admissions due to the limited number of patients (Fig. 2).

The baseline characteristics for all patients and each subgroup are presented in Table 1. Majority of patients were <1 year old (74.1%), with 12.1% admitted to hospital emergency room (ER), while 0.9% were admitted to the ICU. Among the children, pneumonia (7.2%) was the most common comorbidity followed by low-birth weight/premature baby (3.5%). Common procedures were oxygen therapy (44.2%) and mechanical ventilation (3%). Most patients (95%) had laboratory testing and chest x-ray (86.5%). Overall, the average length of hospital stay was 6.9 days with only minor variations across the age groups. The mean total health care cost was JPY 365,583 (USD $3344). Hospitalization of infants <1 year of age incurred higher costs of JPY 420,146 (USD $3843) with DPC costs of JPY 318,713 (USD $2915).

**Table 1:**
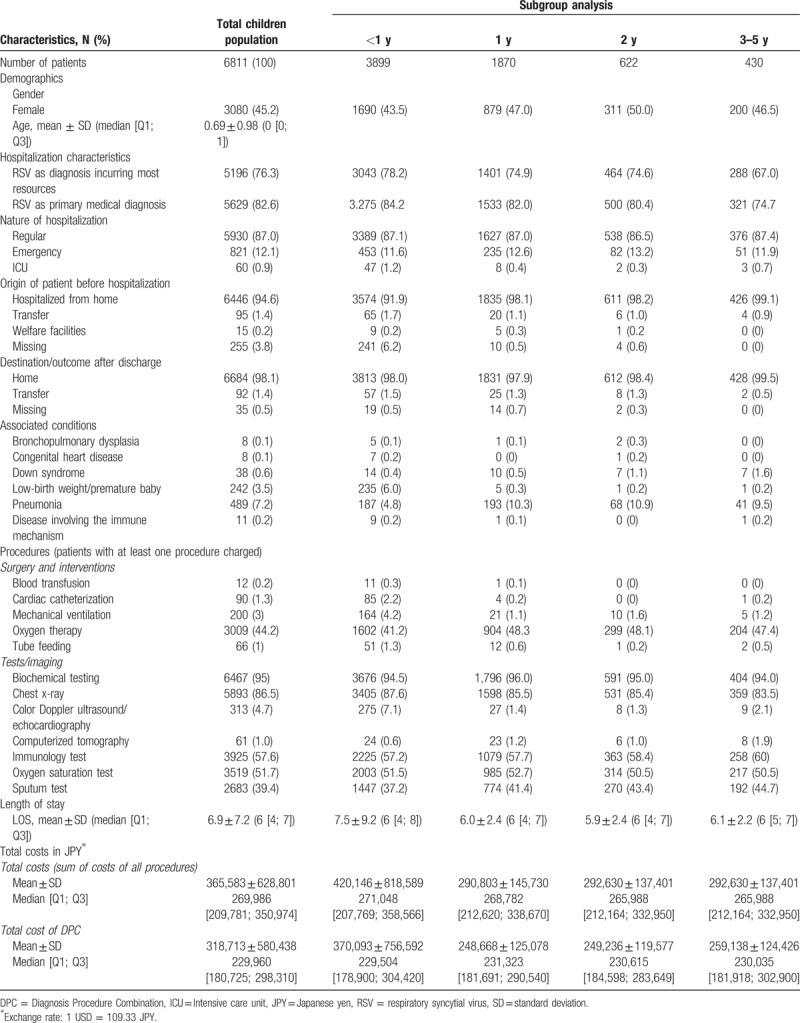
Characteristics of included patients with RSV.

The results of SEM analysis are summarized in Table 2 and the results revealed that infants <1 year of age had higher direct costs but lower indirect costs through lengths of stay resulting in insignificant total effect. The cost of hospital stay was USD $316 per day. As expected, ICU stays were significantly higher in cost (USD +$4951) than routine hospital stays. The ER hospitalizations had a direct positive impact on costs (USD +$458 per day) but apparently shortened LOS and therefore the indirect effect was USD −$242 per day. The small number of infants admitted from welfare institutions resulted in a lower cost (USD −$377 per day).

**Table 2:**
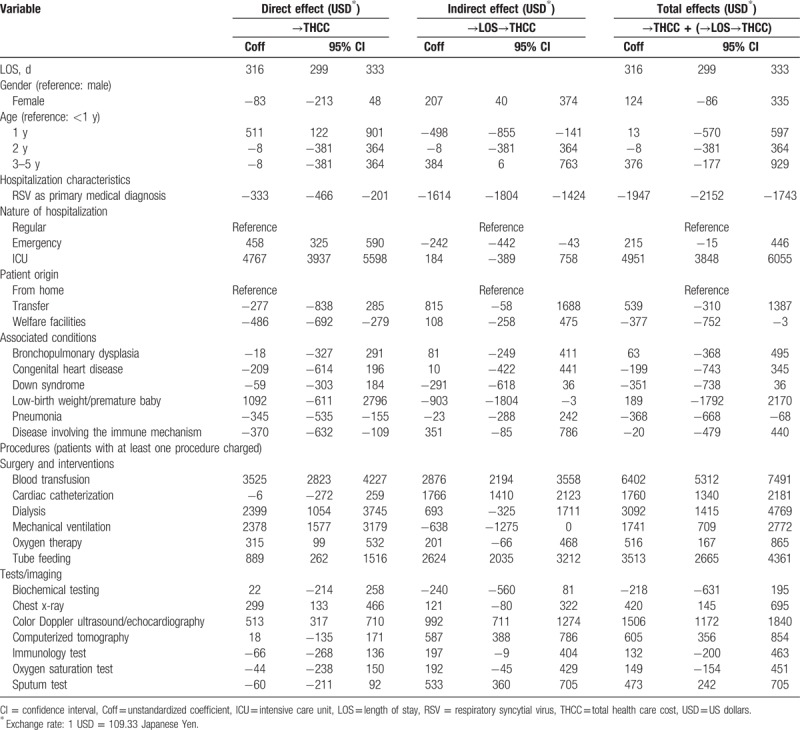
Results of the standard linear structural equation modeling for total costs of hospitalizations: direct, indirect, and total effects.

In general, RSV infection as the primary diagnosis reduced the total cost by USD −$2152 mainly through a decrease in LOS. Among comorbidities, low-birth weight/premature infant had a very high albeit insignificant effect on costs. A significantly negative overall relation was found with pneumonia (USD −$368 per day).

The greatest cost-drivers in terms of medical procedures were: blood transfusion (USD +$6402), tube feeding (USD +$3513), dialysis (USD +$3092), cardiac catheterization (USD +$1760), and mechanical ventilation (USD +$1741). The costs associated with the diagnostic imaging procedures of echocardiography (USD +$1506) increased overall health care costs significantly.

## 4 Discussion

This retrospective cross-sectional study utilized a national administrative database to measure the direct health care costs associated with RSV-related hospitalization. We have demonstrated that in Japan 74% of RSV-related hospitalization occur in children who are <1 year old. We further found that costs are lower when RSV is the primary diagnosis. Clearly, physicians code serious manifestations of the RSV infection as the primary diagnosis. The average LOS was 6.9 days in the overall sample with 7.5 days within the age group <1 year of age. The hospital stay cost on an average USD $316 per day. LOS in Japan appears to be longer compared to that reported for other countries. In Malaysia for instance, 4 days were reported as the average LOS.^[37]^ Similarly, in the United States, the average LOS in hospital is 3.5 days for infants with bronchiolitis, a common clinical presentation of RSV infection,^[38]^ while it was 5.7 days for Canada.^[39]^ Interestingly, in Japan long hospital stays have been reported for other medical conditions in particular mental disorders.^[40]^ Nevertheless, the total health care costs in Japan are significantly lower than in the United States or Canada; the costs for RSV-related hospitalization were JPY 365,583 (USD $3344) in our study which is nearly 78% lower than that reported for the US hospital cost (USD $14,832).^[15]^

Lower health care costs in Japan compared to the United States for other conditions such as leukemia^[41]^ have also been reported or antimicrobial-resistant infections.^[42]^ The main reason is due to differences in health care systems between the 2 countries. For instance, the health care market in the United States is largely unregulated in terms of price setting with much higher prices for both health care and therapeutic services.^[43,44]^

We found the most common procedure, oxygen therapy, administered to 44.2% of the patients increased the total costs by USD $516 per hospitalization. Although only performed for severe infection cases, blood transfusions and tube feedings were identified as the most expensive medical procedures in the hospital.

Our findings of a high cost burden associated with ICU or ER admissions when compared with routine hospitalizations are consistent with previous studies and underscore the importance of preventing ICU admissions whenever possible.

Compared to other pediatric diseases, RSV seems to incur higher costs as well. A recent study analyzed the disease burden of measles in Japan and reported total costs for inpatients of USD $2525^[45]^ which is significantly lower than the results we found in this study.

### 4.1 Implications

We have identified considerable economic burden of RSV infection in Japan which warrants implementation of strategies to manage the impact of the disease. However, since most children affected with RSV infection are usually healthy prior to hospitalization, control strategies targeting only high-risk children will have a limited effect on the overall disease burden of RSV infections.^[2]^ Therefore, the development of a vaccine to immunize infants against RSV infection should be a high public health priority.^[46]^ At present, several live-attenuated and chimeric virus vaccine approaches are being developed. Despite the challenges faced with stabilizing the genetics as well as striking a balance between attenuation (safety) and immunogenicity, much progress has been achieved in developing a recombinant RSV vaccine.^[47]^

Another potential option available for patients at high risk of infection includes pharmacotherapy with drugs such as palivizumab that is used to prevent the infection. In a recent study, treatment of infants at 32 to 34 weeks gestational age with palivizumab was found to be a more economical strategy and improved quality adjusted life years compared to no prophylactic treatment.^[15]^ In other subpopulations, however, the calculated incremental cost-effectiveness ratio ranged from USD $44,774 to $464,476 which is not considered to be cost-effective in various health care systems. The availability of more effective treatments than pavilizumab that reduce severity and shorten the duration of infection could avert the need for hospitalization or reduce the level and duration of hospital care required.

### 4.2 Limitations

There are several limitations to our study. Firstly, this analysis is based on a 1-year database. Consequently, we were unable to capture possible changes due to prescribing behavior changes and any treatment guideline modifications implemented over time. Secondly, due to the inherent limitations of the database, potentially useful information that might explain associated costs was lacking. For instance, we could not retrieve hospital ID numbers, which may have assisted in identification of heterogeneity between hospitals as well as patient characteristics such as region, social status, professional position, clinical severity of their disease, etc. Nevertheless, we examined all available patient characteristics (such as age, gender, and relevant comorbidities) that could be retrieved from the database. Finally, bias may have been introduced from the current DPC system that allows hospitals to select for the diagnosis that is incurring the most medical resource utilization as the principle diagnosis. In general, patients with comorbidities will receive a higher reimbursement if hospitals choose comorbidities as the primary diagnosis.

## Acknowledgment

The authors are grateful to Dr Negar Jamshidi for editing and proofreading the manuscript.

### 4.3 Declarations and ethical approval

This study was conducted in line with the guidelines provided by Johnson & Johnson and approved by the Janssen Approval Committee. This was a retrospective study carried out using hospital claims data from the Medical Data Vision database; the authors were not involved in the collection of this data. Patients were informed that their data would be used for research purposes (opt-out system) and their data were de-identified before addition to the research database. Retrieval of the data from this database occurred in an unlinked fashion. As the data had been anonymized, the Ethical Guidelines for Epidemiological Research (Ministry of Education, Culture, Sports, Science and Technology, and Ministry of Health, Labour and Welfare of Japan), which require ethics approval and informed consent, are not applicable to this study. Based on the Ethical Guidelines on Biomedical Research Involving Human Subjects (Ministry of Education, Culture, Sports, Science and Technology, and Ministry of Health, Labour and Welfare of Japan), pharmacoepidemiologic studies conducted on medical databases constitute research carried out on pre-existing material and information, that did not require any interventions or interactions with patients. For such studies, including this study, obtaining written informed consent from patients is not compulsory.

## Author contributions

**Conceptualization:** Jörg Mahlich.

**Formal analysis:** Rosarin Sruamsiri.

**Validation:** Hiroshi Kubo.

**Writing – original draft:** Jörg Mahlich.
